# 3D printing scanning electron microscopy sample holders: A quick and cost effective alternative for custom holder fabrication

**DOI:** 10.1371/journal.pone.0182000

**Published:** 2017-07-28

**Authors:** Gabriel N. Meloni, Mauro Bertotti

**Affiliations:** Departamento de Química Fundamental, Instituto de Química, Universidade de São Paulo, São Paulo—SP, Brazil; Institute of Materials Science, GERMANY

## Abstract

A simple and cost effective alternative for fabricating custom Scanning Electron Microscope (SEM) sample holders using 3D printers and conductive polylactic acid filament is presented. The flexibility of the 3D printing process allowed for the fabrication of sample holders with specific features that enable the high-resolution imaging of nanoelectrodes and nanopipettes. The precise value of the inner semi cone angle of the nanopipettes taper was extracted from the acquired images and used for calculating their radius using electrochemical methods. Because of the low electrical resistivity presented by the 3D printed holder, the imaging of non-conductive nanomaterials, such as alumina powder, was found to be possible. The fabrication time for each sample holder was under 30 minutes and the average cost was less than $0.50 per piece. Despite being quick and economical to fabricate, the sample holders were found to be sufficiently resistant, allowing for multiple uses of the same holder.

## Introduction

Scanning Electron Microscopy (SEM) is a powerful analytical tool for studying chemical composition, material structure and morphology on the submicron level and thus has been widely used to characterize new materials with a broad applications spectrum. [1,2] Although SEM is a well-established technique for characterizing nanomaterials, such as nanoparticles, it has been used lately to characterize samples that have macroscopic physical dimensions, such as electrode surfaces, ultramicroelectrodes (UME), nanoelectrodes and nanopipettes. Although other techniques, such as Atomic Force Microscopy (AFM) [3], Transmission Electron Microscopy (TEM) [4] and Scanning Transmission Electron Microscopy (STEM) [5] can be used to characterize those samples, SEM is still the “first line of defense” as it is widely available, can give results fairly fast, and can still give important information such as the outer body dimensions of electrodes,[6–9] imperative for a precise calculation of the radius of glass in UMEs. [10] Although some of those parameters can be estimated by electrochemical methods, [11–14] without the knowledge of the precise geometry of the samples one can, misleadingly, interpreter the electrochemical results wrongly and assume false characteristics for the electrode in hand. [15]

The characterization of nanoelectrodes, UMEs and nanopipettes with SEM requires the use of especial custom-made sample holders that can accommodate the macroscopic body of the electrodes and still allow the nanometer features to be studied. Despite looking at first hand as a simple problem to solve, the manufacturing of specialized sample holders can be a time demanding and costly process, requiring the use of specialized tools and trained staff. In that sense, 3D printers can be powerful tools, speeding up the process and decreasing costs by closing the gap between the end user and manufacturing process. Although there are several 3D printing techniques available, Fused Deposition Modeling (FDM) 3D printers are finding more space on the 3D printing community, mainly due to the decrease in price of the printers, low cost and wide variety on the printing material (thermoplastic polymer filament) and increased resolution, now on the orders of tens of micrometers of layer height.[16] Not surprisingly, the use of 3D printers for fabrication of custom parts for research laboratories has been reported with increased frequency.[17–23]

Herein an alternative approach for fabricating custom SEM holders using FDM 3D printers and free 3D Computer Aided Design (CAD) software is presented. The fabrication of custom sample holders with a cost and time effective technique, such as 3D printing, can benefit a broad range of research subjects being of especial interest for samples that cannot be accommodated on standard sample holders. Although the fabricated SEM holders herein presented are designed aiming at the characterization of ultramicroelectrodes and nanoelectrodes for electrochemical applications, the fabrication method developed (and the 3D printed holders) can be applied in a large variety of samples, including nanostructured materials, as demonstrated further. The proposed fabrication method and the application of low cost SEM sample holders is also an interesting alternative for research and teaching centers.

## Materials and methods

All sample holders were designed according to the specific need using the free 3D CAD software 123D design (Autodesk, USA). Once finished, the design was imported to a Cliever CL1 Black (Cliever, Brazil) FDM 3D printer using its proprietary software. The printing speed was set to 150% of the maximum printer speed (value not specified by the manufacturer), bed temperature to 65°C and extruder temperature to 210°C for all prints. The printing infill percentage influence on the final printed pieces performance is explored further in the manuscript. All holders were printed from 1.75 mm diameter electrical conductive carbon impregnated polylactic acid (PLA) filament (Proto-pasta, USA). SEM images were acquired using a JSM-7401F FEG-SEM instrument (JEOL, JPN). The electrochemical measurements were performed using an Autolab PGSTAT 128N bipotentiostat (Metrohm Autolab, Utrecht, The Netherlands). All electrodes and nanopipettes were fabricated according to well-known methods described in the literature. [4,24]

## Results

**Fig 1:**
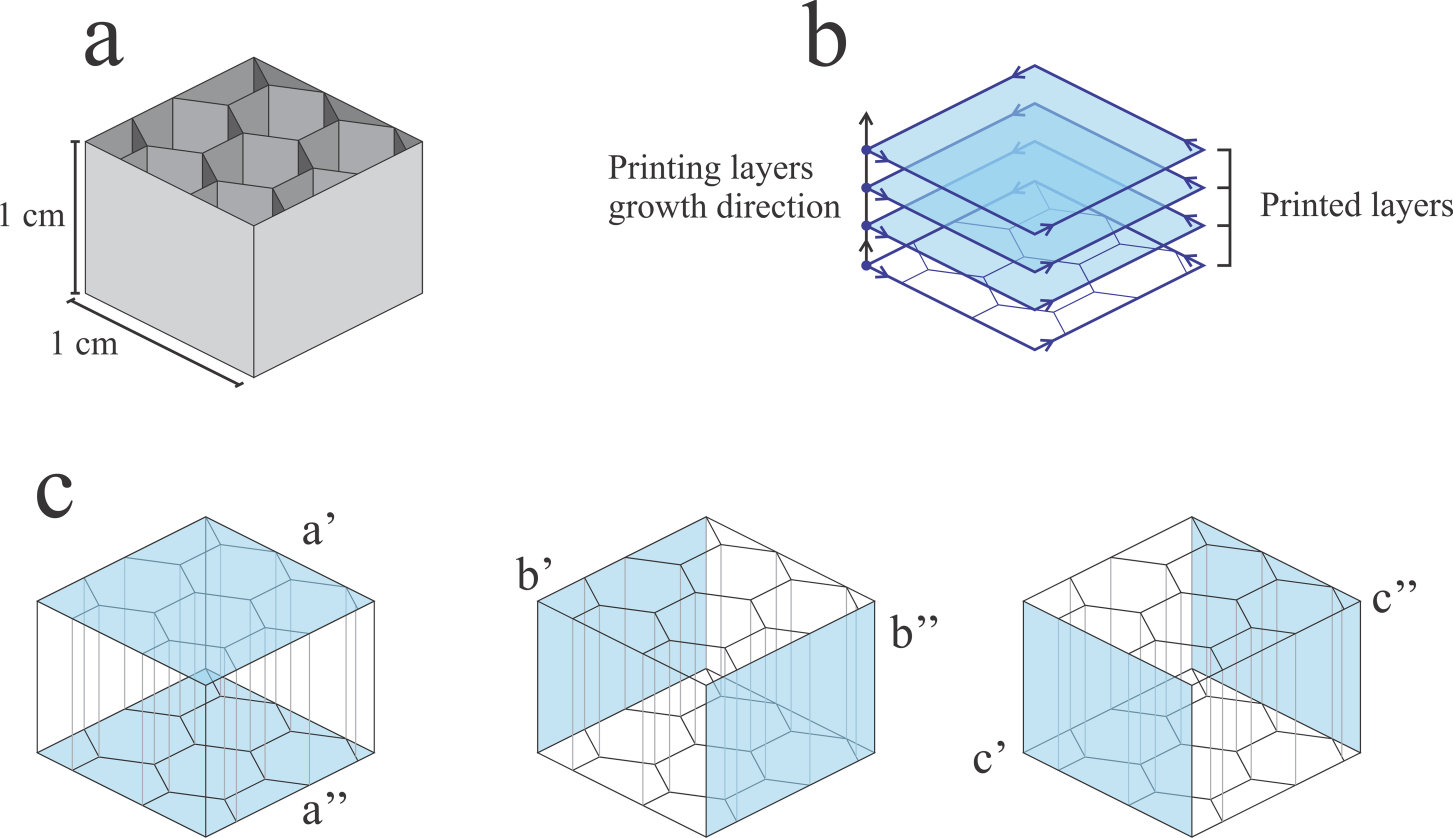
Schematic representation of the 3D printed 1 cm^3^ test pieces. (a) Representation of a test piece with an open top showing the internal infill geometry. (b) Schematic representation of the printing path during the printing of the test pieces, highlighting the staggered layers. (c) Different pairs of opposite faces whose orientation regarding the internal infill geometry are shown in blue.

Despite of the application, a common feature desired in all SEM holders is the ability to conduct, at some extent, electrical current *(i*.*e*. low electrical resistance). This is mainly to create an electrical connection between the sample holder and the SEM chassis which is held at ground potential. The connection avoids the build-up of charge in the sample and sample holder by draining it to ground through the sample holder. This increases the imaging resolution as charged surfaces will create distortions that could yield misinterpretation of the SEM image.[25] To avoid such issues, a commercial carbon impregnated conductive PLA filament was used for printing the sample holders. Although the electrical resistivity of the filament is specified by the manufacture, it regards to the resistivity of a cross section of a solid piece of PLA which is not true for most 3D printed parts by FDM. Most 3D printed parts are hollow or partially filled with a honeycomb-like structure (infill percentage of the 3d printed piece can be adjusted on most printers), as can be seen in Fig 1. This serves two purposes, one is saving printing time and the other is saving printing material. Hence, to proper measure the electrical resistivity, 1 cm^2^ sided cubes were printed with different infill percentages (20, 40 and 100) and used as test pieces.

**Fig 2:**
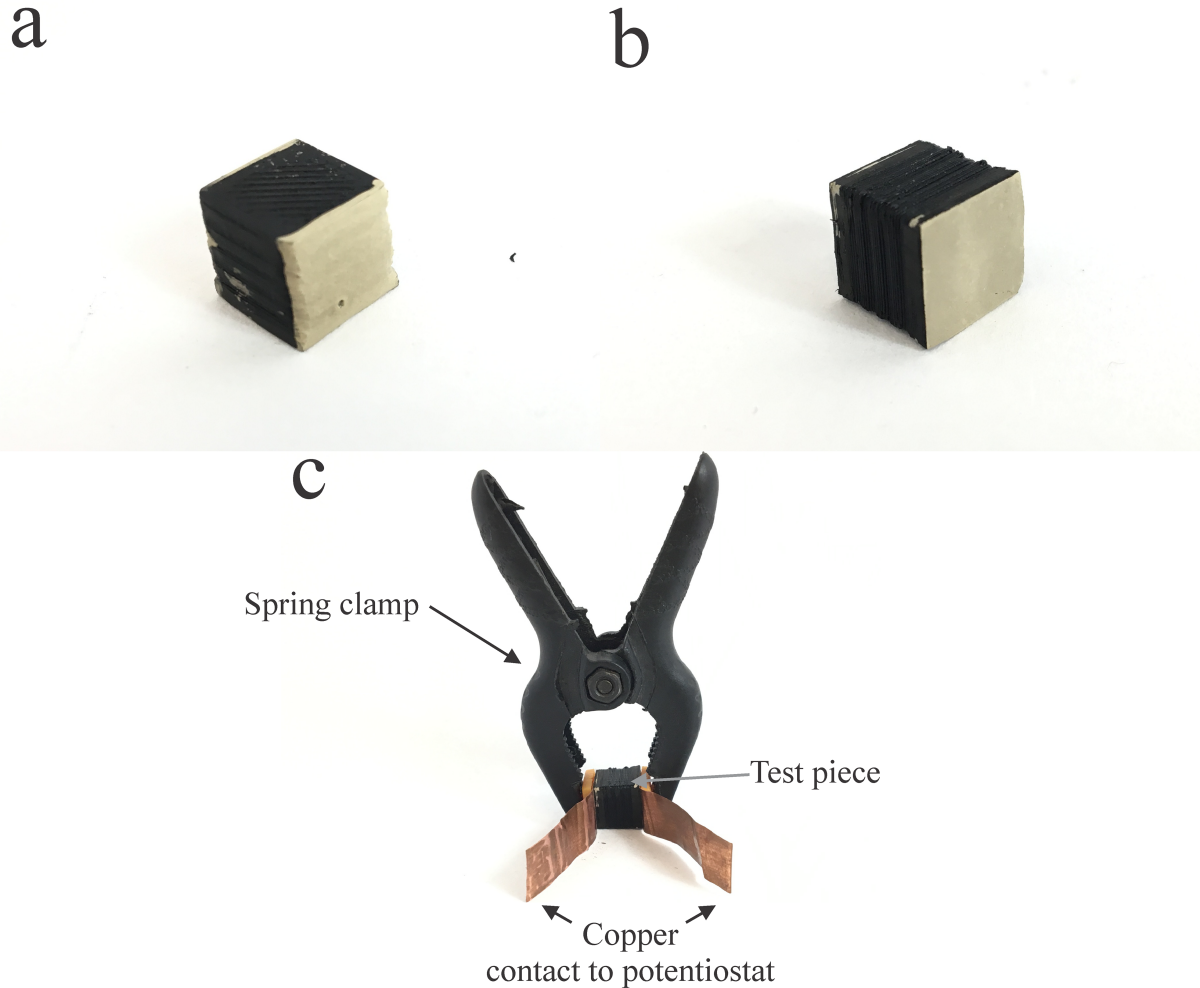
3D printed test pieces with different infill percentages for electrical resistivity calculation showing faces painted with conductive silver ink to avoid contact resistance on (a) faces perpendicular to the printed layers and (b) face parallel to the printing layers. (c) setup for connecting the test pieces to a potentiostat to measure the electrical resistivity. Copper sheets act as a contact between the silver painted faces and the potentiostat. Spring clamp ensures constant pressure on the aces during all the measurements.

To measure the resistivity, two opposite faces of each of the test pieces where coated with a layer of silver conductive ink (Fig 2), reducing the contact resistance for the resistivity measurements. The opposite faces were connected to the leads of a potentiostat and the potential across both surfaces was scanned between 0.2 V and -0.2 V. The slope of the resulting Current *vs*. Potential curve is the inverse of the electrical resistance of the test piece (Ohm’s law) and can be used to calculate its resistivity.

As can be seen by inspection of Table 1, the resistivity of a test piece with a common used infill percentage (20%) changes in a large extent depending on which of the 3 pairs of opposite faces are used in the measurement. The observed difference may rely on the non-uniformity of the 3D printed structure (Fig 1A). Although it is straightforward to see that an increase in infill percentage would result in a decrease in resistivity, as shown in Table 1 for a’ and a” face pairs, one can see that a 100% infill piece not only has a resistivity much higher (around 110 Ω cm) than a solid piece of the conductive polymer (15 Ω cm [26]), but also has a higher resistivity than the one measured between faces b’ and b” and faces c’ and c” of the 20% infill pieces. This is due to the additive fabrication method used, according to which the pieces were fabricated by adding a series of stacked layers as demonstrated in Fig 1B. This results in a material that despite being a solid piece of PLA is still anisotropic due to the poor binding between each of the printed layers. This is less of an issue when the resistivity measurements are taken for faces that are perpendicular to the printed layers (b’/b” and c’/c”), as those are printed as a continue string ensuring good electrical resistivity. One should note that the resistivity of the 3D printed part is an order of magnitude smaller (S1 Fig of the supporting information) than the resistivity of conductive carbon tape commonly used for fixing samples onto sample holders for SEM image.

**Table 1:** Electrical resistivity measured in 3D printed 1 cm^3^ test pieces with different infill percentages. Measurements were taken between different faces of the test cubes.

Infill (%)	Face pairs	Electrical resistivity (Ω cm)
*20*	*a’ and a”*	*129*.*46 ± 0*.*01*
*20*	*b’ and b”*	*84*.*3 ± 0*.*1*
*20*	*c’ and c”*	*61*.*07 ± 0*.*05*
*40*	*a’ and a”*	*114*.*20 ± 0*.*07*
*100*	*a’ and a”*	*109*.*98 ± 0*.*02*

### 3D printed SEM sample holders

**Fig 3:**
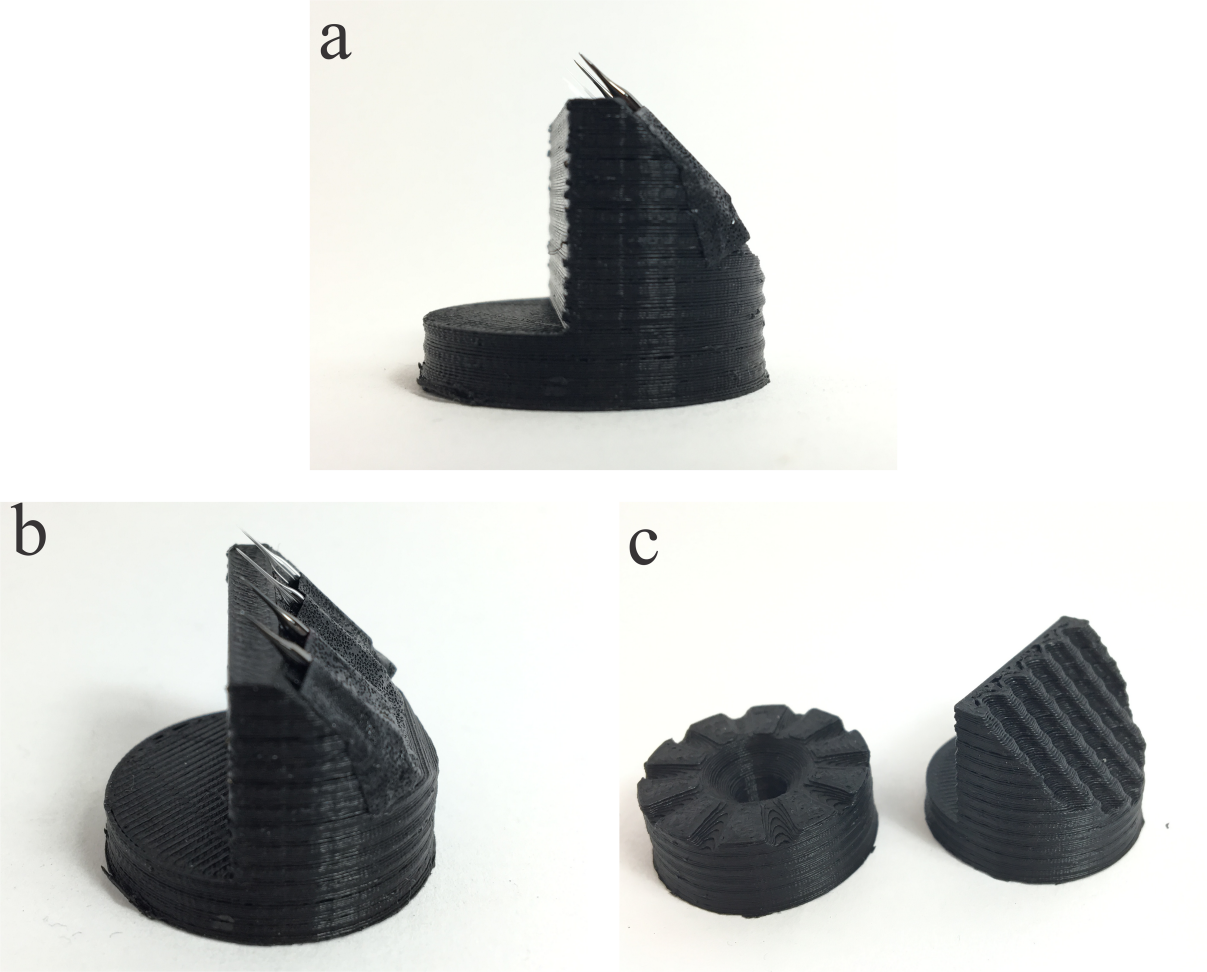
(a) and (b) custom designed 3D printed conductive 45° SEM sample holder for UME and nanopipettes. Nanopipettes attached using conductive carbon tape. (c) Couple of custom designed and 3D printed SEM sample holders showing specific features for holding UMEs and nanopipettes.

Although the 100% infill test piece presented the lower resistivity for measurements taken across faces a’ and a” and a lower resistivity is always a desirable characteristic for SEM sample holders, the printing time and material consumption for printing 100% infill custom sample holders would make this unreasonable. Also, as the binding between layers is not perfect, pockets of air could be trapped between them, which could cause, in the worst case scenario, a violent decompression of the holder when placed in a vented SEM chamber. An easy way around those issues is to design a bottomless sample holder that exposes the infill geometry, thus allowing for the holder to be safely vented inside an SEM chamber. The infill percentage value is a trade between electrical resistivity and printing time and material cost and should be chosen according to the printer used. Accordingly, an infill percentage of 50% was chosen in this work for the 3D printed SEM sample holders. A set of custom made 3D printed SEM sample holders for imaging UME, nanoelectrodes and nanopipettes can be seen in Fig 3.

The 3D printed holders shown in Fig 3 were designed to have specific features that allow for a better positioning and imaging of the electrodes, such as the grooves that allow for precisely positioning each individual electrode. Although a metal sample holder can be made and machined to have the same features, its fabrication can hardly be achieved in a research laboratory as it demands the use of a machine shop and results in time consuming and expensive processes. The total printing time for each of the holders shown in Fig 3 was less than 30 minutes, which is a short period of time to fabricate a finished and functional product.

**Fig 4:**
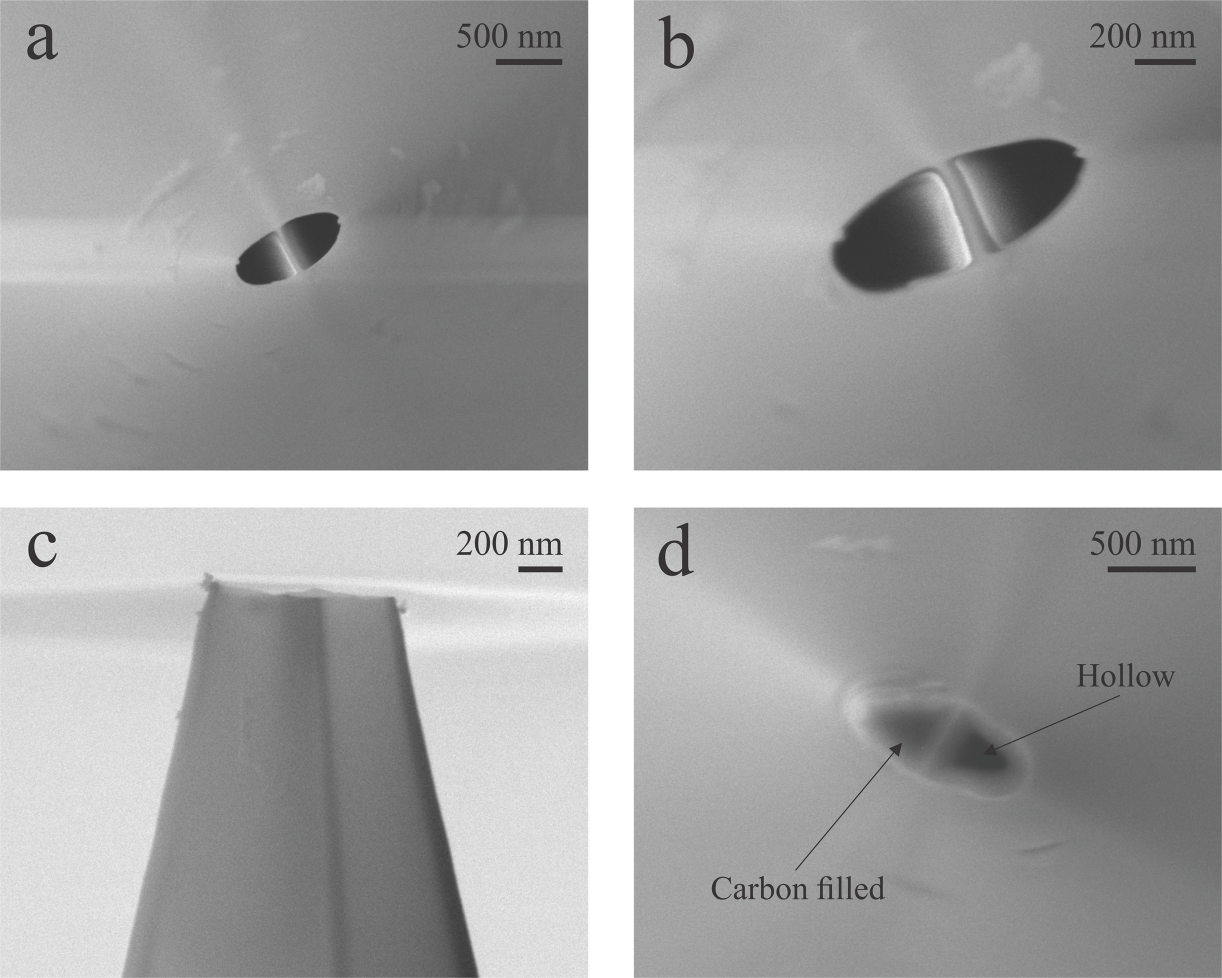
SEM images of nanopipettes and nanoelectrodes. (a) and (b) top view of a double barrel pipette showing both openings unblocked. (c) Side view of a double barrel pipette. (d) Top view of a dual-function pipette based electrode showing selective carbon deposition in only one barrel. Accelerating voltage = 5 kV.

The fabricated sample holders were used to image double barrel (teta) nanopipettes intended to be used as multifunctional probes for electrochemical imaging experiments. This multifunctional nanoprobes can be applied on single crystal analysis, [6] single cell [27–30] and single nanoparticle imaging, [31–33] among others. The knowledge of the critical dimensions of those nanopipettes is crucial for precise characterization,[4,34] being SEM one of the few alternatives for acquiring such information. The nanopipettes were imaged using the 45 degree 3D printed sample holder (Fig 3) and the obtained images can be seen in Fig 4.

**Fig 5:**
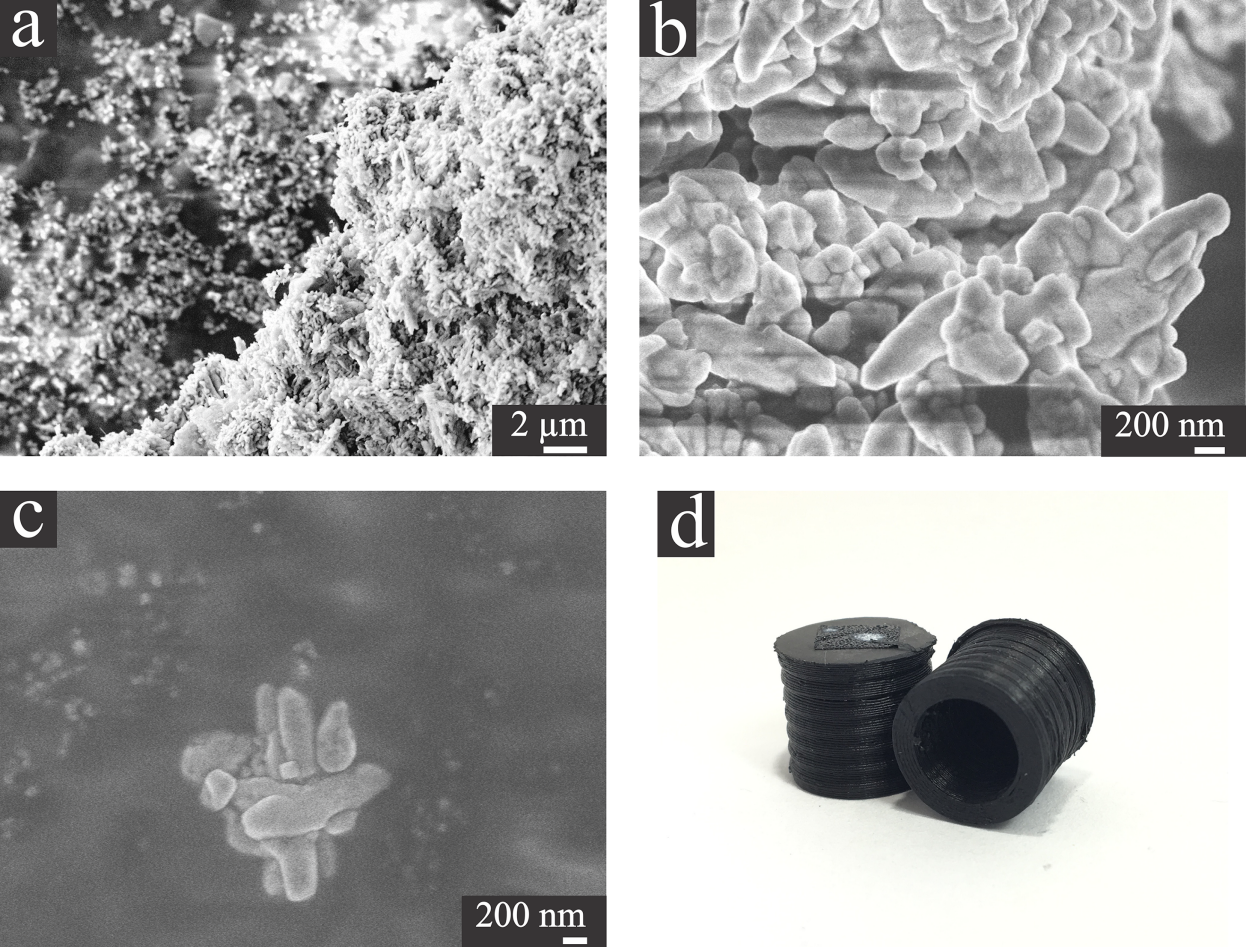
(a) and (b) SEM images of a 300 nm alumina powder used for polishing electrodes at different magnification. (c) Close-up of a single particle cluster showing the details of the particle surface. (d) 3D printed SEM stub sample holders used for acquiring the alumina powder images. Accelerating voltage = 3kV.

Even though the samples are not conductive (Fig 4A, 4B and 4C) and were not coated with a conductive layer, clear images of the electrodes with resolution in the nanometer range were obtained using the 3D printed sample holders with minimal charging effects. The pipette opening dimensions and the overall tip geometry can be easily measured from the acquired images, allowing for a precise electrochemical calculation of the pipette opening radius as described further. The selective deposition of pyrolytic carbon in only one of the barrels of a double barrel pipette, during the fabrication of a dual function nanoelectrode, [35] can also be clearly seen in Fig 4D. The low resistivity of the printed holders allow not only for electrodes to be imaged but also nanostructured materials. Despite not being the focus of this manuscript, as it normally does not require customized sample holders, the characterization of nanomaterials can also be performed using the 3D printed sample holders, even in non-conductive samples, such as alumina powder, as shown in Fig 5. Despite the presence of some charging effect (more significant in the highest alumina density sites, as seen in Fig 5B), which is expected when imaging non-conductive and uncoated substrates, they still present a reasonable resolution and the size and shape of the alumina particles can be easily extracted from them.

### Nanopipette radius calculation

**Fig 6:**
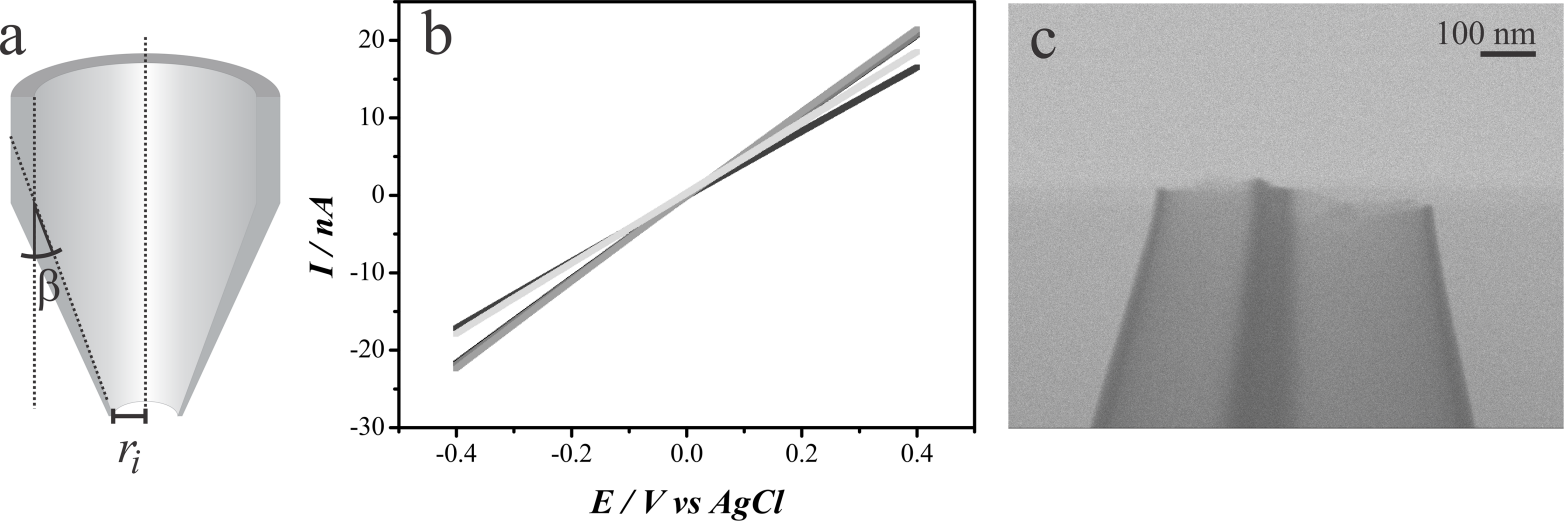
(a) Representation of a cross section of a nanopipette showing the opening radius and the inner semi cone angle, β. (b) Polarization curves recorded with 5 different nanopipettes in a 0.1 mol L^-1^ KCl solution using a pair of AgCl coated silver wires as Quasi-reference counter electrodes (QRCE). Scan rate = 0.1 V s^-1^. (c) Transmission-SEM image of a nanopipette. Accelerating voltage = 15 kV.

It is largely accepted that the radius of nanopipettes can be electrochemically calculated from the resistance to the ionic flow across the pipette opening when a bias potential is applied between two reference electrodes submerged in an electrolyte solution, one placed inside the pipette barrel and the other in the bulk solution. Although this is true, the resistance to ionic flow is not only a function of the pipette radius, it is also a function of the conductivity of the electrolyte solution and the pipette inner semi cone angle (Fig 6A). Eq 1 describes the relationship between those parameters and the pipette radius [4].

$$R_{p}=\frac{1}{K\;\pi \;r_{i}\;tan\beta }+\frac{1}{4\;K\;r_{i}}$$ (1)

Where *R*_*p*_ is the resistance to ionic flow across the pipette opening, *K* is the electrolyte solution conductivity, *r*_*i*_ is the pipette opening radius and β is the inner semi cone angle.

The resistance across the pipette opening is easily calculated by recording a polarization curve. For such, the pipette was filled with 0.1 mol L^-1^ KCl solution and a AgCl coated silver wire was inserted inside the barrel to act as a quasi-reference counter electrode (QRCE). Another AgCl coated silver QRCE was placed in the bulk of the solution. Using a potentiostat, the potential across those two QRCE was scanned between -400 mV and 400 mV at 100 mV s^-1^. The resulting polarization curves for 5 different nanopipettes can be seen in Fig 6B. The slope of the linear fit of the data points is the inverse of the resistance across the pipette opening (*R*_*p*_) (Ohm’s law) and can be used to calculate the nanopipette radius using Eq 1. The inner semi cone angle value cannot be calculated by electrochemical methods, hence the need for using imaging techniques, such as SEM, for proper characterizing nanopipettes. Although an estimative of the inner semi cone angle can be made from the outer semi cone angle using SEM images of the side of nanopipettes (Fig 4C), this would not be very precise as the inner and outer pipette wall do not taper parallel to each other, hence a technique capable of imaging the inner wall of the nanopipette, such as electron transmission technique, is better suited for acquiring this information.[4]

Despite TEM is the most appropriate technique for imaging the inner features of a nanopipette, the macroscopic nature of the nanopipettes body poses as an obstacle as imaging such sample would require custom made holders or time demanding sample preparation procedures such as Focused Ion beam (FIB) milling. As TEM and FIB are not as commonly available as SEM, one alternative electron transmission technique for acquiring images of the inner portion of a nanopipette is Transmission-SEM (T-SEM) which, usually, requires the use of an especial sample holder to acquire transmission images from a SEM equipment. [36] Due to the electron transparency nature of the nanopipette walls (only a couple nanometers thick), T-SEM images with an adequate resolution were obtained by simply laying the nanopipettes over a regular, “stub” (Fig 5D), sample holder. Fig 6C shows a T-SEM image acquired using a 3D printed SEM holder where the internal features of the nanopipette (such as the wall thickness and the inner semi cone angle) can be easily seen and measured. Using the values obtained from Fig 6B and 6C (β = 8.5°), the nanopipette barrel radius can be calculated as ranging between 108 nm and 126 nm, averaging at 117 nm. The values obtained are expected according to the fabrication procedure and are in accordance with SEM and T-SEM images. The difference among the obtained values is not unusual for such fabrication procedure, hence the need for proper characterization of the pipettes. [34]

### 3D printed sample holders durability

Despite being 3D printed out from a thermoplastic polymer, the 3D printed sample holders were found to be extremely durable and able to be reused several times and for different samples. The irregular surface of the printed sample holders (due to the presence of build lines, typical of FDM 3D printing) limits the adhesion of carbon and copper conductive tapes, which are commonly used to fix samples to holders, making it easy to remove them completely from the holders without leaving much residue. Although soluble in acetone [37], the PLA holders were able to endure an overnight acetone bath without presenting much damage other than partial delamination of the first printing layers (data not shown), demonstrating that they can be cleaned with acetone prior to reuse. No apparent damage was observed by interaction with the electron beam during or after the imaging experiments, even when using higher accelerating voltages (15 kV) necessary for the T-SEM experiments. Granting their durability, the 3D printed holders are much softer than any other metal fabricated sample holder and care against mechanical damage should be taken.

## Conclusion

Using commercial 3D printers, commercial carbon impregnated PLA filament and free 3D cad software, we were able to fabricate custom designed fully functional SEM sample holders for high resolution imaging of nanopipettes and nanoelectrodes. Several different sample holders were constructed by using such fast and simple process, each with a specific use and design feature. The conductive nature of the printing material and the chosen printing parameters allowed the use of 3D printed sample holders for imaging nanostructured materials with satisfactory resolution. Although the initial cost of acquiring a 3D printer can be high, the low price of the printing material result on sample holders that cost (on average) less than $0.50 in material (S2 section of the supporting information), making this an attractive alternative. Despite been made out of PLA, the printed holders were found to be surprisingly durable and able to be re-used several times even after cleaning with diluted acetone solution.

## Supporting information

S1 Fig Images from 123D design free cad software of the designed sample holder. (DOCX)
