# Supplementary material for: 3D printing scanning electron microscopy sample holders: A quick and cost effective alternative for custom holder fabrication
Source: PLoS One. 2017 Jul 28;12(7):e0182000. doi: 10.1371/journal.pone.0182000 (PMC5533330; doi:10.1371/journal.pone.0182000)
Supplement: S1 Fig — (DOCX) [file pone.0182000.s001.docx]

**Supporting information for**

**3D printing scanning electron microscopy sample holders: A quick and cost effective alternative for custom holder fabrication.**

Gabriel N. Meloni* and Mauro Bertotti

Departamento de Química Fundamental, Instituto de Química, Universidade de São Paulo, 05508-000 São Paulo - SP, Brazil

*e-mail: gabriel.meloni@iq.usp.br

*To whom correspondence should be addressed

**Contents Section**

***Commercial Carbon tape conductivity calculation S1***

***Sample holder price calculation S2***

***3D design S3***

**S1 - Commercial Carbon tape conductivity calculation**

As stated by the manufacture, double side conductive carbon tape has a sheet resistivity ranging from 100 kΩ/sq to 250 kΩ/sq. ^1^ Assuming uniformity on the conductive carbon layer, the electrical resistivity (ρ) can be calculated from the sheet resistivity by multiplying the later by the thickness of the sheet. With a layer thickness of 125 µm, the electrical resistivity calculated ranges from 1250 Ω.cm to 3125 Ω.cm, which is higher than the resistivity found for the 3D PLA pieces.

**S2 - Sample holder price calculation**

The 3D printed sample holders used in the SEM imaging and shown on Fig. 3 of the main manuscript had a diameter of 25 mm and an infill of 50%. Although they differ in height and geometry, their mass averaged at 4.1 g. With the retail price (at the time of this manuscript preparation) of $49.99 USD for a 500 g spool of conductive PLA, each holder would have an average material cost of $0.41 USD.

**S3- 3D design**

**
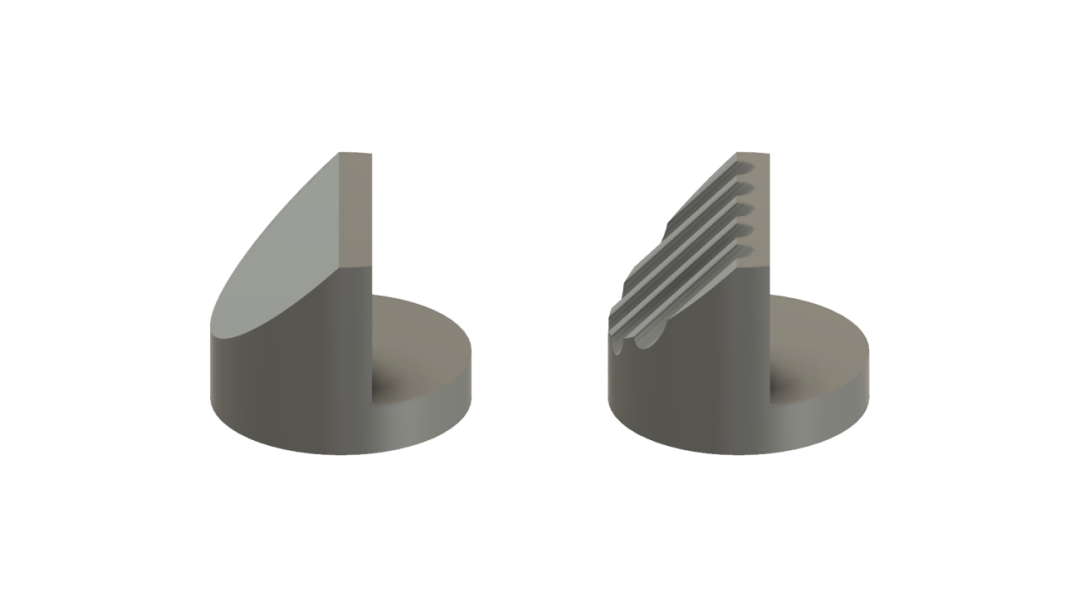

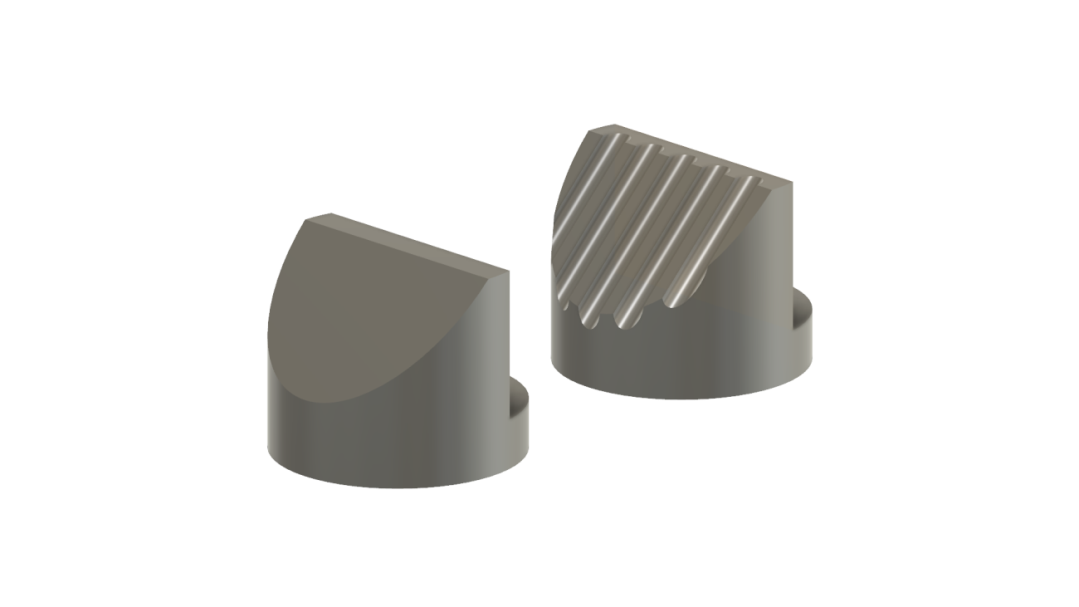
**

**Figure S1.** Images from 123D design free cad software of the designed sample holder.

**References**

1. Conductive Tabs, Tapes & Sheets Comparison Table, 2016. https://www.tedpella.com/adhesive_html/conductive-tapes-comparison.htm.
